# Studies on Biological Test of Formulated Diets Supplementation of Vitamin E for the Broodstock of Females Blue Swimming Crab, *Portunus pelagicus* (Linnaeus, 1758)

**DOI:** 10.12688/f1000research.15885.2

**Published:** 2019-01-03

**Authors:** Efrizal Efrizal, Indra Junaidi Zakaria, Rusnam Rusnam, Suryati Suryati, Nofa Yolanda

**Affiliations:** 1 Departement of Biology, Faculty of Math and Science, Andalas University, Padang, West Sumatra, 25163, Indonesia; 2 Agricultural Engineering, Faculty of Agriculture, Andalas University, Padang, West Sumatra, 25163, Indonesia; 3 Faculty of Phamacy, Andalas University, Padang, West Sumatra, 25163, Indonesia

**Keywords:** Blue swimming crab, female broodstock diet, vitamin E

## Abstract

**Background:** Currently, great progress in the artificial propagation of commercially important   portunid crabs of the genus *Portunus* has been achieved, and various methods have been adopted in mass-scale hatchery activities. This study analyzed the biological testing of formulated diets with different dose supplementations of vitamin E for the broodstock of female blue swimming crabs, *P. pelagicus *(Linnaeus, 1758)

**Methods:** Female crab samples were collected from the coastal region of Padang, West Sumatra. The method used in this study was completely randomized design, with four treatment regimens (n=5 crabs each) of dietary vitamin E (0, 300, 600, and 900 IU/kg formulated diets).

**Results: **The results show that the supplementation of vitamin E in the formulated diet had a significant effect (P <0.05) on the absolute weight growth, carapace length and carapace width.

**Conclusions:** Supplementation of vitamin E on in formulated diet causes broodstock blue swimming crab molting, with a percentage value of 40–80% on day 20 and 20% on day 30, with a 100% survival rate.

## Introduction

The market share of the crab, *Portunus pelagicus*, at the international level from year to year tends to increase, with the main markets in Singapore, Japan, the Netherlands, France, USA, Taiwan, Hong Kong, Korea, etc ^1– 4^. The high market value of, and high demand for, crabs in the global market provide a strong stimulus for their intensive culture. Although the mass production of blue swimming crab seeds was first reported by the Research and Development Center for Oceanology (RDCO)-LIPI in Indonesia ^5^, its culture industry has not developed as expected. A shortage of seeds, lack of suitable feed, lack of an appropriate culture system and poor management are recognized as the major bottlenecks in the development of the crab culture industry in this country. *P. pelagicus* seeds were previously caught in the wild, causing an insufficient seed supply for commercial firms and recreational fisheries. Currently, great progress in the artificial propagation of commercially important portunid crabs in the genus *Portunus* has been achieved and various methods have been adopted for mass-scale hatchery activities. However, farming this species has always been limited by the seasonal availability of spawning crabs from the wild and their inability to mature and spawn in culture ponds. The second major problem associated with the blue swimming crab culture is the high larval mortality during the zoeal and megalopa stages. Thus, it has become necessary to biologically test the supplementation of vitamin E for the formulated diets of broodstocks of females blue swimming crabs, *Portunus pelagicus* (Linnaeus, 1758).

## Methods

### Location and parameters

The study was conducted at the Fish Seed Center Beaches (BBIP) Teluk Buo, Fish Seed Center (BBI) Bungus, of the city of Padang, and the Laboratory of Animal Physiology, Department of Biology, Faculty of Math and Science, Andalas University, Padang, West Sumatra. A total of 70 female crabs were collected on January 2018; 20 were females at stage II of ovarian maturity, which were selected for study. The female crab samples, with mean body weigth (BW) of 158.15 g, carapace length of 57.27 mm and carapace width of 123.54 mm were collected from the coastal region of Padang, West Sumatra, and randomly placed in four concrete tanks (200 × 100 × 100 cm). A total of five units from each tank were placed in a plastic box (45.5 × 32.5 × 16.5 cm) at a maximum density of one crab per box. The tanks were provided with a sand substrate layer approximately 15 cm thick with adequate aeration ^2, 6, 7^. The crabs were maintained a monitored water depth of 25–30 cm with salinity of 29–32 parts per thousand (ppt), pH of 7.26 to 8.00, temperature of 26–28°C, and DO of 6.15–7.45 ppm. Each crab was provided with a shelter made of PVC pipe, 13 cm in diameter and 40 cm in length, to serve as a refuge during molting. 

### Supplementation

Dietary treatments, with supplementation of vitamin E (Nutrimax ™Vitamin E-Water Soluble), were fed daily at 3% of the biomass (1700–1800 hours), and uneaten food was removed every morning. The completely randomised design method with four treatments and (n=5 crabs per treatment) replications of dietary vitamin E was used in this study. The different feed groups were P0 (diet 1, 0 IU/kg formulated diet), P1 (diet 2, 300 IU/kg formulated diet), P2 (diet 3, 600 IU/kg formulated diet), and P3 (diet 4, 900 IU/kg formulated diet). Formulated diet ^8^ is a modified formulation for the broodstock of the mud crab, *Scylla serrata* ^9^. Blue swimming crab broodstock fed diets P0, P1, P2 and P3 were initially fed a natural diet (fresh bivalve molluska + sardinella fish; 1:1) and were gradually acclimatized to the formulated diet until the 10th day of culture. Daily feeding rates were 10% of broodstock biomass for natural food, and 3% for formulated diet (diet 1). Diets were fed twice daily at 1700 and 1800 h, with 40% of the ration given in the afternoon and the remaining 60% in the evening. Excess diet was monitored and feeding rates were adjusted accordingly. Molting and mortality were recorded daily.

### Measured parameters

The Absolute weight growth was calculated as follows: AWG = *WG _f_ – WG _o_*, where AWG is weight gain (g), *WG _f_* is the final size/weight (g), and *WGo* is the weight of crab at the start of experiment (g). The Absolute carapace length was calculated as follows: ACL = *CL _f_ – CL _o_*, where ACL is carapace length gain (mm), *CL _f_* is the carapace length of the crab at the end of experiment (mm), and *CL _o_* is the carapace length of crab at the start of experiment (mm). The Absolute carapace width was calculated as follows: ACW = *CW _f_ – CW _o_*, where ACW is carapace width gain (mm), *CW _f_* is the carapace width of the crab at the end of experiment (mm), and *CW _o_* is the carapace width of crab at the start of experiment (mm). The carapace length, carapace width, percentage of molting and survival rates were measured as described previously ^7, 8, 10^. Weights were measured to 0.01 g on the electronic balances (BL3200H-SHIMADZU). The water quality parameters that were monitored daily were temperature (°C), salinity (ppt), pH, and water depth (cm) while dissolved oxygen (ppm) and CO _2_ (ppm) thrice weekly using a maximum-minimum thermometer, hand-held Atago refractometer model 8808, Thermo Orion Benctop pH meter models 410 A plus, weighted line, YSI oxygen meter model 57, and APHA ^11^, respectively.

### Statistical analysis

Results were given as the means ± SE. The biological test data (absolute weight growth, absolute carapace length, absolute carapace width and survival rate) were tested using one-way ANOVA and Duncan’s multiple range test (p<0.05) to compare the mean differences among the treatments ^12^ were performed using SPSS software (version 19.0 for Windows; SPSS Inc., Chicago, IL). The standard error of each parameter was determined. The data on percentage of molting female broodstock are shown in tables and graphs and then analyzed descriptively.

## Results

### Molting female broodstock

Growth in the mother crab is a good measure of the weight gain, carapace length and carapace width within a certain time after the process of molting occurs. This study shows that each of the different dietary administrations elicited a positive response in terms of the percentage of female broodstock undergoing the molting process. In total, 40–80% of females fed diet 2 and 3 had molted by day 20, but only 20% of females receiving diet 4 had molted by day 30. For those receiving diet 1, the female broodstocks did not molt until maintenance on days 40 ( Table 1).

**Table 1.** Percentage of female broodstock blue swimming crabs, *P. pelagicus* (Linnaeus, 1758), that molted with a formulated diet of vitamin E supplementation at different doses.

Treatment	Replications	Percentage of molting female broodstock				
		0 days	10 days	20 days	30 days	40 days
P0 (diet 1)	1	II	II	II	III	III
	2	II	II	III	III	IV
	3	II	II	II	III	III
	4	II	III	III	III	IV
	5	II	II	II	III	III
	Total	0.00	0.00	0.00	0.00	0.00
	Average	0.00	0.00	0.00	0.00	0.00
P1 (diet 2)	1	II	III	IV *	IV	V
	2	II	III	III *	IV	V
	3	II	III	IV	IV *	IV
	4	II	III	III *	IV	V
	5	II	III	IV *	IV	V
	Total	0.00	0.00	400.00	100.00	0.00
	Average	0.00	0.00	80.00	20.00	0.00
P2 (diet 3)	1	II	III	IV *	IV	V
	2	II	III	III *	IV	V
	3	II	III	IV *	IV	V
	4	II	III	III	IV *	IV
	5	II	II	III	IV	IV
	Total	0.00	0.00	300.00	100.00	0.00
	Average	0.00	0.00	60.00	20.00	0.00
P3 (diet 4)	1	II	II	III	IV	IV
	2	II	III	III	IV *	IV
	3	II	III	IV *	IV	V
	4	II	II	III	IV	V
	5	II	III	IV *	IV	V
	Total	0.00	0.00	200.00	100.00	0.00
	Average	0.00	0.00	40.00	20.00	0.00

*Molting broodstock female. P0 (diet 1), 0 IU/kg formulated diet (control); P1 (diet 2), 300 IU/kg formulated diet; P2 (diet 3), 600 IU/kg formulated diet; P3 (diet 4), 900 IU/kg formulated diet. Gonad maturity stages (GMS) based on Efrizal ^2^ (II: Ovaries are light yellow/orange, III: Ovaries are yellow/orange, large and nodulated, IV: Ovaries are dark yellow/orange and V: Ovaries are light yellow, tans or yellow-orange but not bunched up).

### Absolute weight growth

Growth in absolute weight is a measure of the weight difference of a female reached within a certain time period compared to her weight at the beginning of the period. The average weight and absolute weight gain of female parent crabs, *P. pelagicus* (Linnaeus, 1758), with different dietary treatments are presented in Table 2. Growth in absolute weight is a measure of the weight difference a female reached within a certain time period compared with a weight at the beginning of the period. The average weight and absolute weight gain of female parent crabs, *P. pelagicus* (Linnaeus, 1758), with different dietary treatments are presented in Table 2. Females fed all formulated diets (1, 2, 3 and 4) were likely to increase their absolute weight during the maintenance period of 0 to 40 days. The results ( Table 2) show the highest absolute value of the average weight (45.38 g) was obtained in those receiving diet 2, compared to diet 1 (12.69 g), diet 4 (28.52 g), or diet 3 (32.96 g); with ANOVA showing significant differences (P<0.05). Duncan’s test revealed further significant differences (P <0.05) between the treatments of diet 1 and diet 2; and diet 3 and diet 4, while the treatments of diet 2 and diet 3; and diet 3 and diet 4 did not differ significantly(P>0.05).

**Table 2.** Average and absolute weight (g) of female broodstock blue swimming crabs, *P. pelagicus* (Linnaeus, 1758), with a formulated diet of vitamin E supplementation at different doses.

Sampling (days)	Treatment (n=5 per group)			
	P0 weight (g)	P1 weight (g)	P2 weight (g)	P3 weight (g)
0	158.12 ± 30.05	158.15 ± 26.91	158.19 ± 25.43	158.14 ± 24.34
10	159.53 ± 30.10	160.14 ± 26.89	159.70 ± 25.64	159.69 ± 24.38
20	162.02 ± 30.01	165.08 ± 26.62	163.10 ± 25.54	162.60 ± 24.26
30	166.18 ± 30.00	194.62 ± 31.96	183.74 ± 24.39	176.92 ± 20.37
40	170.81 ± 30.01	203.53 ± 27.87	191.15 ± 28.36	186.68 ± 26.98
AWG	12.69 ± 1.62 ^a^	45.38 ± 3.54 ^b^	32.96 ± 6.72 ^bc^	28.53 ± 6.16 ^c^

Mean values within a given column with different superscripts were significantly different (P<0.05), Values are means ± standard errors (SE). AWG, absolute weight growth; P0 (diet 1), 0 IU/kg formulated diet (control); P1 (diet 2), 300 IU/kg formulated diet; P2 (diet 3), 600 IU/kg formulated diet; P3 (diet 4), 900 IU/kg formulated diet.

### Absolute carapace length

Absolute carapace length is calculated from the difference between the parent crab carapace length at a certain period of time and the carapace length at the beginning of the study. The use of different diets caused relatively large changes in the growth of the absolute carapace length during the maintenance period of 40 days, which range between 1.00 and 6.23 mm ( Table 3), with ANOVA showing significant differences (P<0.05). Table 3 shows accretions of the highest absolute carapace length obtained in the treatment of diet 2 (6.23 mm) compared to treatments of diet 1 (1.00 mm), diet 3 (3.76 mm) and diet 4 (3.76 mm). Duncan’s post hoc test reveals significant differences (P <0.05) between the treatments of diet 1 and diet 2, and diet 3 and diet 4, whereas diet 3 and diet 4 treatments are not significantly different (P> 0.05).

**Table 3.** Average carapace length (mm) and absolute carapace length (mm) of female broodstock blue swimming crabs, *P. pelagicus* (Linnaeus, 1758), with a formulated diet of vitamin E supplementation at different doses.

Sampling (days)	Treatment (n=5 per group)			
	P0 carapace length (mm)	P1 carapace length (mm)	P2 carapace length (mm)	P3 carapace length (mm)
0	56.53 ± 3.21	56.50 ± 2.80	57.98 ± 2.15	58.07 ± 2.37
10	56.53 ± 3.21	56.50 ± 2.80	57.98 ± 2.15	58.07 ± 2.37
20	56.53 ± 3.21	57.10 ± 2.81	58.38 ± 1.97	58.47 ± 2.16
30	57.53 ± 3.21	61.30 ± 3.29	61.16 ± 1.83	60.98 ± 1.44
40	57.53 ± 3.21	62.73 ± 0.72	61.74 ± 2.01	61.74 ± 2.28
ACL	1.00 ± 0.00 ^a^	6.23 ± 0.72 ^b^	3.76 ± 0.63 ^c^	3.67 ± 1.46 ^c^

Mean values within a given column with different superscripts were significantly different (P<0.05). Values are means ± standard errors (SE). ACL, absolute carapace length; P0 (diet 1), 0 IU/kg formulated diet (control); P1 (diet 2), 300 IU/kg formulated diet; P2 (diet 3), 600 IU/kg formulated diet; P3 (diet 4), 900 IU/kg formulated diet.

### Absolute carapace width

Based on the measurements ( Table 4), artificial feeding with a supplementary dose of 0 IU of vitamin E (Control) provides an added value mean carapace width that is relatively low (1.00 mm) compared to artificial feeding at a dose of 900 IU of vitamin E (7.40 mm), 600 IU (7.41 mm) and vitamin E 300 IU (13.06 mm); with an ANOVA showing significant differences (P<0.05). Similarly, post hoc Duncan’s test shows significant differences (P<0.05) between the treatments of diet 1 and diet 2, while the treatments of Diet 2 with Diet 3 and diet 4 showed no significant differences (P>0.05).

**Table 4.** Average carapace width (mm) and absolute carapace width (mm) of female broodstock blue swimming crabs, *P. pelagicus* (Linnaeus, 1758), with a formulated diet of vitamin E supplementation at different doses.

Sampling (days)	Treatment (n=5)			
	P0 (diet 1)	P1 (diet 2)	P2 (diet 3)	P3 (diet 4)
0	121.39 ± 7.27	121.91 ± 5.08	126.04 ± 5.06	124.82 ± 3.92
10	121.39 ± 7.27	121.91 ± 5.08	126.04 ± 5.06	124.82 ± 3.92
20	121.39 ± 7.27	122.51 ± 5.10	126.44 ± 4.85	125.22 ± 3.72
30	122.39 ± 7.27	131.31 ± 7.83	132.66 ± 4.52	129.44 ± 2.80
40	122.39 ± 7.27	134.97 ± 5.96	133.45 ± 4.54	132.22 ± 5.75
ACW	1.00 ± 0.00 ^a^	13.06 ± 2.48 ^b^	7.41 ± 2.53 ^ab^	7.40 ± 3.56 ^ab^

Mean values within a given column with different superscripts were significantly different (P<0.05). Values are means ± standard errors (SE). ACW, absolute carapace width; P0 (diet 1), 0 IU/kg formulated diet (control); P1 (diet 2), 300 IU/kg formulated diet; P2 (diet 3), 600 IU/kg formulated diet; P3 (diet 4), 900 IU/kg formulated diet.

### Survival rate

The results show that feeding different diets to the female parent crabs during the maintenance period of 40 days in controlled cultivation containers, had a high survival rate (100%) for all treatments ( Table 5). A high survival rate is due to maintenance in a controlled container, where there were no deaths in the female parent crabs. This likely occurred because the water quality (physical and chemical factors) during the study was in the viable range for living crabs ( Table 6).

**Table 5.** Percentage of average survival rate (%) of female broodstock blue swimming crabs, *P. pelagicus* (Linnaeus, 1758), with a formulated diet of vitamin E supplementation at different doses.

Sampling (days)	Treatment (n=5)			
	P0 survival rate (%)	P1 survival rate (%)	P2 survival rate (%)	P3 survival rate (%)
0	100 ± 0.00	100 ± 0.00	100 ± 0.00	100 ± 0.00
10	100 ± 0.00	100 ± 0.00	100 ± 0.00	100 ± 0.00
20	100 ± 0.00	100 ± 0.00	100 ± 0.00	100 ± 0.00
30	100 ± 0.00	100 ± 0.00	100 ± 0.00	100 ± 0.00
40	100 ± 0.00	100 ± 0.00	100 ± 0.00	100 ± 0.00
H	100 ± 0.00 ^a^	100 ± 0.00 ^a^	100 ± 0.00 ^a^	100 ± 0.00 ^a^

Mean values within a given column with different superscripts were significantly different (P<0.05). Values are means ± standard errors (SE). H, survival rate; P0 (diet 1), 0 IU/kg formulated diet (control); P1 (diet 2), 300 IU/kg formulated diet; P2 (diet 3), 600 IU/kg formulated diet; P3 (diet 4), 900 IU/kg formulated diet.

**Table 6.** The water quality of the maintenance media for female broodstock blue swimming crabs, *P. pelagicus* (Linnaeus, 1758), with a formulated diet of vitamin E supplementation at different doses.

Water quality parameter	Range
Salinity (ppt)	29.0–32.0
Temperature (°C)	26.0–28.0
pH	7.26–8.00
O _2_ (ppm)	6.15–7.45
CO _2_ (ppm)	3.78–6.10
Water depth (cm)	25.0–30.0

ppt, parts per thousand.

Spreadsheet containing data associated with this study Data include time of molting, carapace width and carapace length. Click here for additional data file. Copyright: © 2019 Efrizal E et al. 2019 Data associated with the article are available under the terms of the Creative Commons Zero "No rights reserved" data waiver (CC0 1.0 Public domain dedication).

## Discussion

Molting is the process of the replacement of old shell with new shell, and is a cycle that occurs in all types of arthropods, ranging from insects to crustaceans. It is important for growth, reproduction and metamorphosis ^13^. Fujaya *et al.* ^14^ explained that the activities of seeding, growth and soft shell crab production become more efficient when the mechanisms of reproduction and growth of the animals in culture are understood. In this experiment, it is clear that an artificial diet at a dose of vitamin E 300 IU/kg (diet 2), 600 IU/kg (diet 3) and 900 IU/kg affects the physiological process of the test animal known as molting (ecdysis) ( Table 1). Fujaya *et al.* ^14^ and Kuballa *et al*. ^13^ mention that several factors stimulate the process of molting in crustaceans, namely, external information from the environment such as light, temperature and food availability. Studies using vitamin E as a nutritional source for crustaceans have been performed by several previous investigators, including Winestri *et al*. ^15^ on *Scylla paramamosain* and Nasution *et al*. ^16^ on *Macrobrachium rosenbergii*. These researchers mentioned that vitamin E-supplemented feed gives the best results for the growth of mangrove crabs and the fecundity of prawns.

The high absolute weight gained in the treatment with diet 2 compared to diet 1 and diet 4 is due to the improvement in nutritional quality by supplementation with tocopherols (such as vitamin E) being balanced with the composition of other nutrients in the composition of the parent artificial diet. Cahu *et al.* ^17^ found that vitamin E plays a role in the improvement of the reproductive performance of crustaceans. The addition of 300 mg/kg vitamin E in the diet of crustacean broodstocks is considered to increase the potential of reproduction that affects the growth of the absolute weight of aquatic animals ^18^. Furthermore, Izqiuerdo *et al*. ^19^ explain that a lack of vitamin E results in the compromised development of reproductive organs toward mature gonads.

The difference in carapace length in those receiving diet 2 from those receiving diets 1, 3 and 4 is significant due to the higher percentage of females that undergo the molting process with diet 2 (80% at day 20 and 20% at day 30) compared to those with diet 1 (0% at day 40), diet 3 (60% at day 20, 20% at day 30), and diet 4 (40% at day 20 and 20% at day 30). According to Fujaya *et al.* ^14^ and Kuballa *et al*. ^3^, the growth and reproduction of crabs and other crustaceans are closely related to the molting cycle phase and control stimulation (ecdysteroids) in its hemolymph. Furthermore, four phases are described in the molting cycle; intermolt, premolt (preparation for molting), ecdysis and postmolt (recovery from molting). During intermolt, exoskeletons formed perfectly, and the crustaceans accumulated stored calcium and energy. Premolt begins when the old exoskeleton begins to separate itself and a new epidermis begins to form. The newly formed exoskeleton is larger and is still pale and soft. Postmolt is the new exoskeleton’s hardening process. The low accretion of mean carapace length of diet 1 (1.00 mm) compared to diets 2, 3 and 4 is due to the crab not experiencing replacement of the skin (molting). The added carapace length is allegedly due to changes in the shape of the carapace. Sulaeman and Hanafi ^20^ mention that carapace length changes in the female broodstocks that do not undergo molting are caused by changes in the curvature of the back shell margin, where the eggs mature making the rear carapace increasingly convex. According to the results reported for observations of the mud crab, *S. serrata*, accretion is obtained when the carapace length ranges between 0 and 3 mm during a maintenance period of 35 days.

The low-growth carapace width of the female parent in those receiving diet 1 compared with the other diets happened because the treatment did not induce molting in the maintenance period from 0 to 40 days. The increasing carapace width is supposed to have occurred because of the shape changes in the carapace, which can be seen in the change in curvature of the back of the carapace. The greater the weight of the parent crab, the more convex the carapace became. The absolute changes in carapace width are relatively large in the artificial diet treatment with a dose of 300 IU vitamin E (diet 1). The diet treatment causes the broodstock females to undergo molting. Maheswarudu *et al*. ^20^ and Kuballa *et al*. ^13^ state that the differences in growth in the cultivation of crabs are caused by several factors, such as feeding, age, baseline weight, space and other factors. Furthermore, they explains that the more feed consumed, the larger the crab will become and the more frequent the replacement of the skin. Crab shell replacement last occurred between 17 and 26 days, and every crab molted, and increased by one-third of its original size. According to Fujaya *et al*. ^14^ and Kuballa *et al.* ^13^, the molting process that occurs in crustaceans in principle is caused by two factors: internal and external (such as feed, light and other factors). Thus, both factors will affect the brain and stimulate the Y organs to produce hormone molting. Molting is controlled by a steroid circulating in the exoskeleton hemolymph that stimulates the synthesis of new and the regeneration of integument lost before molting ^13^. Ecdysteroid is a crab molting hormone, which is secreted by the organs in the form of ecdysone-Y ^21^. Inside the hemolymph, the hormone is converted into an active hormone, 20-OH-ecdysone hydroxylase, which is present in the epidermis organs and other body tissues ^13, 21^.

Water quality is one of the limiting factors in the crab culture system. The crab is active entirely in water, where it carries out functions such as respiration, excretion of wastes, feeding habits, growth and reproduction. According to Habashy and Hassan ^22^, the required water quality parameters for the maintenance of crustaceans are salinity, temperature and pH ^23^. Salinity affects the osmotic pressure of the water. The higher the salinity, the greater the osmotic pressure ^23^. For the cells of all animal organs to function properly, the cells must receive a liquid medium with the appropriate composition and concentration of ions. It appears that like other marine invertebrates, a marine crustacean living in water that is isotonic with the blood has a water medium and generally has a similar ionic composition, but this differs slightly; a large difference can even occur between the ionic composition of the fluid in the cell (haemolymph) with the sea water medium. Therefore, osmoregulation is required to ensure that the intracellular and extracellular composition and concentration of the ionic liquid remains normal or balanced ^21, 24– 26^. Additionally, Stiarto *et al*. ^27^ and Silvia *et al*. ^28^ reported that hyperosmoregulation in crustaceans requires energy in the form of protein or lipids ^29, 30^. Table 6 shows that the water salinity range of the maintenance media during the observation period was between 29.0 and 32.0 ppt. Salinity is still within the range that is highly favorable to the survival and reproductive activities of the crabs ^17, 31, 32^. Environmental temperature also affects the growth and survival of aquatic organisms. Nearly all aquatic organisms are very sensitive to abrupt environmental temperature changes. At 5°C ambient temperature abrupt changes can cause stress or even death in some types of cultured organisms ^12^. In this study, the average water temperature during the observation ranged between 26.0 and 28.0°C. The temperature is in the support range for the activities of life, growth and reproduction of the crab ^33, 34^. The pH of the water media during the study ranged from pH 7.26 to 8.00. This shows that the pH of the water was maintained within the range of neutral and slightly alkaline, and was thus suitable for living crabs. According to the Ministry of Environment in Anonymous ^35^, water with a pH range of 6.5–8.5 has a considerable potential for the development of aquaculture in terms of productivity. Taslihan *et al*. ^36^ stated that alkaline waters would be more productive than acidic waters.

Dissolved oxygen plays an important role in the life of all living organisms. However, there is a difference between the oxygen required by living organisms in terrestrial and aquatic organisms. Terrestrial organisms consume oxygen contained in the air, while aquatic organisms take in dissolved or bound oxygen. The oxygen requirements for every type of aquatic biota differ depending on the species toleration of the rise and fall of oxygen. In general, all types of cultured organisms (fish, shrimp, crabs, clams, and sea cucumbers) are unable to tolerate fluctuations in oxygen that are too extreme ^14^. Dissolved oxygen (O _2_) was relatively high in this experiment, ranging between 6,15 to 7,56 ppm. The amount of oxygen that must be maintained to ensure a good life for aquatic organisms is not less than 3 ppm. If the oxygen content drops to less than 2 ppm, some kinds of crustaceans will be under pressure and will even die ^37, 38^. According to Millamena and Quinitio ^9^. Crab cultivation requires dissolved oxygen at a concentration greater than 4 ppm. Although the role of carbon dioxide (CO _2_) is considerable for living aquatic organisms, very excessive levels would interfere, and are even toxic to cultivated biotas. The tolerance of each biota varies depending on its type and body size, and is generally no more than 15 ppm. A concentration of over 25 ppm of carbon dioxide is dangerous for cultured organisms ^39, 40^ because its presence in the blood can inhibit the binding of oxygen by hemoglobin. Furthermore, Dodd *et al*., ^33^ Long *et al*., ^41^ and Landes and Zimmer ^42^ reported that acidification caused by CO _2_ has been found to strongly affect calcification rates, animal behavior, predator foraging behavior and the avoidance of predators by prey. Carbon dioxide measurements ( Table 6) in the study ranged between 3.78 and 6.10 ppm. Boyd and Tucker ^43^ explain that free carbon dioxide is good for the crustacean no higher than 12 ppm and must not be less than 2 ppm.

## Conclusions

The conclusions that can be drawn from the results of this experiment are as follows: (1) Diet 2, with supplementation of 300 IU/kg vitamin E formulated diet, provided for the highest absolute weight gain (45.38 g), absolute carapace length (6.23 mm), and absolute carapace width (13.06 mm); (2) supplementation of the formulated diet with 300 IU/kg vitamin E in the formulated diet also causes broodstock blue swimming crab molting, with a percentage of 40–80% on day 20 and 20% on day 30; and (3) a survival value of 100% was obtained for all treatments during the 40 days maintenance period.

Further studies are needed to observe and analyze the effects of formulated diet supplementation of vitamin E on the incubation period and reproductive performance of female broodstock blue swimming crabs, *P. pelagicus* (Linnaeus, 1758) in mass production.

## Data availability

The data referenced by this article are under copyright with the following copyright statement: Copyright: © 2019 Efrizal E et al.

Data associated with the article are available under the terms of the Creative Commons Zero "No rights reserved" data waiver (CC0 1.0 Public domain dedication). 

**Dataset 1. Spreadsheet containing data associated with this study.** Data include time of molting, carapace width and carapace length. DOI: https://doi.org/10.5256/f1000research.15885.d221868 ^44^.
