# Supplementary material for: Studies on Biological Test of Formulated Diets Supplementation of Vitamin E for the Broodstock of Females Blue Swimming Crab, Portunus pelagicus (Linnaeus, 1758)
Source: F1000Res. 2019 Jan 3;7:1780. Originally published 2018 Nov 12. [Version 2] doi: 10.12688/f1000research.15885.2 (PMC6305238; doi:10.12688/f1000research.15885.2)
Supplement: Peer review history

# Peer review: Referee response for version 2

**DOI:** 10.5256/f1000research.19385.r42506

**Published:** 2019-01-10

**Authors:** Zainal Abidin Muchlisin

**Affiliations:** 1 Department of Aquaculture, Faculty of Marine and Fisheries, Syiah Kuala University, Banda Aceh, Indonesia

The authors have made corrections as suggested.

I have read this submission. I believe that I have an appropriate level of expertise to confirm that it is of an acceptable scientific standard.

# Peer review: Referee response for version 1

**DOI:** 10.5256/f1000research.17343.r40547

**Published:** 2018-12-17

**Authors:** Zainal Abidin Muchlisin

**Affiliations:** 1 Department of Aquaculture, Faculty of Marine and Fisheries, Syiah Kuala University, Banda Aceh, Indonesia

Title:

 I suggest to modify "Growth performance, survival rate and moulting frequency of blue swimming crab (Portunus pelagic, Linnaeus, 1978) fed on varying level of vitamin E

 Introduction:

 The introduction is lack of state of the arts of the study on blue swimming crab and vitamin E. Therefore, the author must emphasize and cite the current studies or previous findings by other researchers on blue swimming crab and the role of vitamin E on crab or aquatic organism.

 There was no clear objective of the study. So please state the objective clearly at the end of introduction section.

 Methods:

 Fish Seed Centre Beach is not appropriate term of BBIP., need to change. 

 Where the crab is come from?

 The author should mention whether the feed used is a commercial diet or experimental diet (formulated by researcher for this study). And also the proximate composition of the diets are unclear. So the author must present the composition of the nutrition content (proximate composition) of the tested diet, maybe presented in the table would be better.

 How the procedure to mix the vitamin E with the diet? 

 Existing: The method used in this study was completely randomised design methods with ......"

 Suggestion: The completely randomised design method with  ...was used in this study"

 Results:

 Table 1 is unclear. The author stated table 1 presented the data of percentage of moulting female brood stock. Actually, The percentage value should be ranged 0-100%. But the author provided the value between II - IV. In my understanding this is not percentage data, but this is the maturity stage of ovary. The authors have to clarify.

 The author should mention the results of the ANOVA test whether the experiment gave the significant effect on measured parameters or not. And the author also have to mention briefly the differences values (data) among the treatments (based on Duncan's multi rage test). Please see superscript after the value.

 Discussion:

 In general the discussion is already acceptable, but maybe the author has to cite more references to support the discussion, especially from reputable related  journals.

 Conclusions:

 The conclusion should be clear and strong if possible. Your conclusion should be focused on the objective.

I have read this submission. I believe that I have an appropriate level of expertise to confirm that it is of an acceptable scientific standard, however I have significant reservations, as outlined above.

# Response

**Published:** 2019-01-03

**Authors:** Efrizal Efrizal

**Affiliations:** Andalas University, Indonesia

1. **Comment 1**: ***Answer:*** **Title: **I have read carefully, the suitable title from my manuscript is“Studies on Biological Test of Formulated Diets Supplementations of Vitamin E for the Broodstock of Females Blue Swimming Crabs, *Portunus pelagicus* (Linnaeus, 1758)”
2. **Comment 2**: ***Answer: ***Introduction: We have been searching other reference, we could not find the latest research of blue swimming crab and vit E.
3. **Comment 3:** ***Answer: ***Methods: have been modified.
4. **Comment 4**: ***Answer*** **: Results**: see Table 1. The red supercript shows the percentage of molting broodstock female.

          ANOVA: We have stated the significant differences in the treatment. Different supercript

          letter means significant differences.

# Peer review: Referee response for version 1

**DOI:** 10.5256/f1000research.17343.r40546

**Published:** 2018-12-04

**Authors:** Ambok Bolong Abol-Munafi

**Affiliations:** 1 Institute of Tropical Aquaculture, Universiti Malaysia Terengganu, Kuala Nerus, Malaysia

Major English revision needed trough out the manuscript.

 Title

 The title did not reflect the contents of the manuscript. The term of 'Biological Test' did not appear in the text.

 Introduction

 Should highlight the common parameters for biological test. Authors should also discuss the functions of vit E in fishes and the type of vit E that commonly used. 

 Methods

 The methodology should provide:

- Information on the quality of base diet, especially on the protein and fat contents.
- Brief explanation on how to identify the ovarian maturity stages of live samples
- Explanation on type of vitamin E used and how it apply to the feed. Why IU unit was used instead of mg/g diet.
- Only 20 stage II broodstocks were used. One crab per replicate (5 replicate per treatment). Not enough for statistical analysis

Results

- Molting

Why molting occurred exactly on 10, 20, 30 and 40 days. Actual data on molting day should be presented. Table 1 showing more on maturity stages compare to molting.

 Discussions

 Should discuss in detail the role of vit E on molting

I have read this submission. I believe that I have an appropriate level of expertise to confirm that it is of an acceptable scientific standard, however I have significant reservations, as outlined above.

# Response

**Published:** 2019-01-03

**Authors:** Efrizal Efrizal

**Affiliations:** Andalas University, Indonesia

1. **Comment 1:** ***Answer*** :English grammar have been revised. We have send to American Journal Expert (AJE), certificate attached.
2. **Comment 2**: ***Answer***: The biological test in the title have been explained in the method analysis. The biological analysis include absolute weight, absolute carapace length, absolute carapace width, survival rate and molting female broodstock.
3. **Comment 3:** ***Answer***: we have discuss it.
4. **Comment 4:** **Methodology.**

- The quality of base diet not discuss in this manuscript because we would like to publish in the next article.
- The ovary condition can be seen by the pressing of the gap between the back and the abdomen of the crab.
- Explanation on type of vitamin E used and how it apply to the feed. Why IU unit was used instead of mg/g diet.
- We have answer the question from reviewer Hafrijal Syandri **(**Nutrimax ™Vitamin E-Water Soluble **)**
- The IU is an International Unit, usually used to measure fat soluble vitamins including Vitamin E.
- I think 5 replicate per treatment enough. Because the canibal behaviour from the crab. During the experiment we use plastic box. Each plastic box have the maximum density only one crab.

**      5. Comment 5. *Molting. Answer:*** my observation every 10 days.

          Data molting.....see Table 1. Have been presented.

      6. **Comment 6:** ***Answer*** **: Discussion**: Have been discussed detail in the manuscript.

# Peer review: Referee response for version 1

**DOI:** 10.5256/f1000research.17343.r40545

**Published:** 2018-11-16

**Authors:** Hafrijal Syandri

**Affiliations:** 1 Department of Aquaculture, Faculty of Fisheries and Marine Sciences, Bung Hatta University, Padang, Indonesia

**Abstract:**

**In method**: The author did not mention the type of vitamin E used for feed supplements. Please add the type of vitamin E used including the producer of vitamin E or name of the company.

**In results**: The author did not mention the best dose of vitamin E as the diet supplement. Which one the best dose of Vitamin E for absolute growth, carapace length and carapace width.

**Keywords**: please choose the unique words, not similar with the title.

**Methods:**

**Location:** The study was conducted at the Fish Seed Center Beaches (BBIP) Gulf Buo……name of location no need translate in English, example Gulf Buo, must be written " *Teluk Buo*”

**Supplementation**: Dietary treatments, with supplementation of vitamin E, were fed daily at 3%......please add initial weight, length and width of carapace from the broadstock.

 Vitamin E, please state the name of the company…for example Ovaprim, (manufactured for Syndel Laboratories Ltd, 2595 McCullough Rd. Nanaimo, B.C.V9S 4M9 Canada). What type of vitamin E used in the experiment? Liquid or powder?

 The author did not mention the name of balance scale used during the experiment….please state the model of balance, for example ﬁsh were weighed using balance scale (OHAUS model CT 6000-USA).

**Result:**

**Molting female broadstock**: Growth in the mother crab is a good measure…..the word “mother” is not suitable…please change to broadstock.

 Absolute weight growth: The average weight and absolute weight gain of female parent crabs… the word “parent” is not suitable…please change to broadstock.

**Conclusions:**

 The conclusions that can be drawn from the results of this experiment are as follows: (1) Diet 2, …….please rewrite and state only the significant finding of your research.

I have read this submission. I believe that I have an appropriate level of expertise to confirm that it is of an acceptable scientific standard.

# Response

**Published:** 2019-01-03

**Authors:** Efrizal Efrizal

**Affiliations:** Andalas University, Indonesia

1. Comment 1: **Abstract** has been modified. Have been revised, ***Answer*** :Nutrimax ™Vitamin E-Water Soluble; The highest absolute weight gain (45.38 g), absolute carapace length (6.23 mm), and absolute carapace width (13.06 mm) were promoted by supplementation of 300 IU/kg vitamin E in formulated diet; Keywords: Molting, ovary, gonad maturity stages,
2. Comment 2: **Methods** have been modified. Have been revised; ***Answer*** :The study was conducted at *Balai Benih Ikan Pantai *(BBIP) **Teluk Buo**, *Balai Benih Ikan* (BBI) Bungus, Padang; 20 were females at stage II of ovarian maturity, which were selected for study. The female crab samples with mean  body weigth (BW) of 158.15 g, carapace length  of 57.27 mm and carapace width of 123.54 mm were collected from the coastal region of Padang, West Sumatera; Weights were measured to 0.01 g on the electronic balances (BL3200H-SHIMADZU).
3. Comment 3: **Result** **not revised**
4. Comment 4: **Conclusion** : have been revised; ***Answer***: (1) **Fdiet 2** with supplementation of 300 IU/kg vitamin E formulated diet, provided for the highest absolute weight gain (45.38 g), absolute carapace length (6.23 mm), and absolute carapace width (13.06 mm); 2) supplementation of the formulated diet with 300 IU/kg vitamin E in the formulated diet also causes broodstock blue swimming crab molting, with a percentage of 40–80% on day 20 and 20% on day 30; and; (3) a survival value of 100% was obtained for all treatments during the 40 days maintenance period.
